# CNN1 Represses Bladder Cancer Progression and Metabolic Reprogramming by Modulating HIF-1α Signaling Pathway

**DOI:** 10.3389/fonc.2022.859707

**Published:** 2022-07-12

**Authors:** Zheng Zhang, Xiaoqing Li, Shaokun Ren, Wei Zhang

**Affiliations:** ^1^ Tianjin Institute of Urology, Second Hospital of Tianjin Medical University, Tianjin, China; ^2^ Phase I Clinical Trial Department, Tianjin Medical University Cancer Institute and Hospital, Tianjin, China

**Keywords:** bladder cancer, CNN1, glycolysis, HIF-1α, overexpression

## Abstract

Bladder cancer (BC) is the second most common urinary system malignant tumor around the whole world. It has been reported that CNN1 was lowly expressed in BC tissues. However, the mechanisms of CNN1 on BC cells were unclear. Herein, we aimed to probe the specific influences of CNN1 on BC pathogenesis. First, the expression level and prognostic ability of CNN1 in BC patients were surveyed. Then, CNN1 overexpression was executed to exhibit the influences of CNN1 on BC cells. The real-time PCR and Western blotting were employed to detect by the mRNA and protein expression levels. CCK8 assay was utilized to examine cell proliferation, and transwell assay was executed to test cell invasion and migration. The corresponding kits were utilized to detect glucose absorption, lactate secretion, and ATP level. BC cells overexpressing CNN1 were utilized to establish a nude mouse xenograft tumor model, and the tumor volume and tumor weight were detected. Nude mouse tumor tissues were used for immunohistochemical experiments to test the expression levels of Ki-67 and CNN1. The outcomes indicated that CNN1 was significantly lowly expressed in BC tissues and cells. Besides, low expression of CNN1 might be concerned with poor prognosis. Moreover, overexpression of CNN1 repressed the proliferation, invasion, and migration of BC cells. Furthermore, CNN1 overexpression decreased the protein levels of glycolysis-related protein GLUT1 (glucose transporter 1), pyruvate kinase M2 (PKM2), and LDHA (lactate dehydrogenase A). Then, the decreased mRNA and protein levels of HIF-1α and PDK1 were identified after CNN1 overexpression. The *in vivo* assays verified the effects of aberrant expression of CNN1 in mice with BC. In conclusion, these findings suggested that CNN1 might modulate BC progression through activating HIF-1α pathway and CNN1 might be a promising marker for BC diagnosis.

## Introduction

Bladder cancer (BC) is the second most common urinary system malignant cancer around the worldwide (1). The number of patients worldwide has been increasing in recent years (2). In China, the incidence and mortality of BC rank first among urinary system tumors (3). Chemotherapy and surgical resection are the two main therapies for BC (4). Nevertheless, when the cancer progresses to an advanced stage, it is manifested as easy distant metastasis, severe drug resistance, and poor clinical prognosis (the 5-year overall survival rate is only 60%), and the effect is not ideal (5, 6). Due to the lack of prevention, diagnosis, and specific treatment, most patients have reached the advanced stage as soon as they are discovered (7). Therefore, in the face of these urgent challenges, exploring new biomarkers and identifying effective therapeutic targets for BC will help to develop more effective anticancer therapies.

Calponin 1 (CNN1) was first confirmed in chicken gizzards and is one of the regulators of actomyosin contraction (8). Recently, studies have manifested that CNN1 is concerned with the development of various cancers (9). CNN1 is downregulated in uterine leiomyosarcoma (10). It has been reported that CNN1 was lowly expressed in BC, and CNN1 was related to patient survival through bioinformatics (11). *In vitro* CNN1 was a cancer inhibitor gene that serves as an indicator of liver cancer cell migration (12). However, the specific mechanism of CNN1 in BC is still not very clear and remains to be revealed.

As belongs to the family of AGC kinases, phosphorylation of pyruvate dehydrogenase kinase 1 (PDK1) is composed of serine and threonine kinases that show a sequence homology of their catalytic domain correlated to cAMP-dependent protein kinase 1, cGMP-dependent protein kinase, and protein kinase C (13). As reported, PDK1 acted a vital effect in breast cancer development (13). Increased levels of PDK1 have been reported in 45% of patients with acute myeloid leukemia, and a role for PDK1 in ovarian cancer progression has also been proposed (14, 15). PDK1 was repressed in periostin−expressing UMUC−3 cells (16). The mechanism about the essential of PDK1for BC is still unclear.

As it is known to all, hypoxia-inducible factor 1 (HIF-1), an oxygen-sensing transcription factor, decides whether glucose is expended through oxidation or glycolysis (17). A variety of factors in the tumor microenvironment, such as hypoxia (18) and certain metabolites (19), can regulate the hydroxylase activity of PHD2 to restrain HIF-1α degradation, resulting in augmented HIF-1α protein levels and elevated aerobic glycolysis in cancer cells (20). Chen et al. (17) revealed that RNA interference–mediated silencing of HISLA to stable HIF-1α might offer a more viable method to suppress glycolysis of cancer cells. In corpora cavernosum smooth muscle cells, the expression of CNN1 was downregulated by endogenous HIF-1α small interfering RNA under hypoxic conditions (21).

In this article, we explored whether CNN1 influenced the properties and carcinogenesis of BC. It was discovered that CNN1 was lowly expressed in BC cells. Besides, CNN1 had some impacts on BC cell proliferation, invasion, migration, and glycolysis, which might be possibly concerned with HIF-1α pathway. These findings may offer a novel standpoint into BC pathogenesis and suggest a novel therapeutic target or prognostic biomarker for BC.

## Methods

### Bioinformatics Analysis

In this study, the 30 BC patients and 30 normal samples were acquired between 2019 and 2021 from our hospital. In addition, four BC patients (T1, T2, T3, and T4) and four normal samples (N1, N2, N3, and N4) were chosen to analyze the protein level of CNN1. On the basis of The Cancer Genome Atlas (https://gdc-portal.nci.nih.gov/) database and the median expression value of CNN1, BC patients were fallen into high and low expression groups. The log-rank test was utilized to determine the statistical significance. The research involving human tissue was approved by the Medical Ethics Committee of The Second Hospital of Tianjin Medical University.

### Cultivation of BC Cell Lines

Human BC cell lines EJ, 5637, UMUC-3, T24, and J82 and normal control urothelial epithelial cell line SV-HUC were gained from the Chinese Academy of Medical Sciences Shanghai Cell Bank (Shanghai, China). These cells were routinely cultivated in Dulbecco’s minimum essential medium including 10% fetal bovine serum (FBS) and 1% penicillin-streptomycin in a 5% CO_2_ incubator at 37°C.

### Cell Transfection

pcDNA3.1-CNN1 and pcDNA3.1 (vector) were acquired from GenePharma Co. (Shanghai, China). When the cells were attaining 80% mixing, they were transfected into cells utilizing Lipofectamine 2000 reagents (Invitrogen, USA). After that 48 h, the transfection efficiency was determined by Quantitative Reverse Transcription-Polymerase Chain Reaction (qRT-PCR) measurements after 48 h.

### qRT-PCR

Total RNA was extracted utilizing TRIzol (Invitrogen). For the reverse transcription of RNA, PrimeScript RT Reagent Kit (Takara, Kyoto, Japan) was applied. qPCR analysis was executed to detect SYBR Premix Ex Taq™ Kit (Takara) on the ABI 7300HT system. The expression level of CNN1 was normalized against glyceraldehyde-3-phosphate dehydrogenase and relatively quantified utilizing 2^-ΔΔCt^ method. The primers in this study were displayed in **Table 1**.

**Table 1:** The primers utilized in qRT-PCR.

Name	Sequences
CNN1 forward	5'- CCAACGACCTGTTTGAGAACACC-3'
CNN1 reverse	5'- ATTTCCGCTCCTGCTTCTCTGC-3'
GAPDH forward	5'-TGTGTCCGTCGTGGATCTGA-3'
GAPDH reverse	5'-CCTGCTTCACCACCTTCTTGA-3'

### Western Blotting

The total protein was separated by radioimmunoprecipitation assay lysis buffer (Cw Biotech, Bejing, China) including proteinase suppressors. The protein concentration was assessed by bicinchoninic acid (Beijing ComWin Biotech Co., Ltd., Beijing, China) reagent. After that, 20 µg of protein samples was detected by 10% sodium dodecyl sulfate–polyacrylamide gel electrophoresis gels, followed by transfer to a polyvinylidene difluoride (PVDF) membrane. After being blocked with 5% fat-free milk for 1 h, the PVDF membranes were hatched with the antibodies at 4°C overnight. The blots were tested with ECL.

### CCK8

After 48 h of transfection, T24 and UMUC-3 cells were seeded into 96-well plates (1,000 cells per well). The proliferation of T24 and UMUC-3 cells was detected by CCK-8. One hundred microliters of cell suspension was taken, and 1 × 10^3^ cells per well were placed in 96-well plates. After that, 10 μl of CCK8 reagent was added into each well, followed by incubation for 1.5 h at 37°C incubator. The absorbance at 450 nm was detected by a microplate reader.

### Transwell Assays

*In vitro* invasion and migration of T24 and UMUC-3 cells were tested by transwell inserts precoated with or without Matrigel. In brief, 200 μl of serum-free medium including approximately 1 × 10^5^ transfected cells was hatched into the upper chamber of each transwell. Besides, 500 μl of complete medium (including 10% FBS) was put onto the lower chamber and incubated for 24 h at 37°C. Then, the cells were fixed by 4% paraformaldehyde and dried with 0.1% crystal violet for 10 min each. Last, the number of cells was counted at selected randomly five fields utilizing a light microscope at magnification ×200.

### Detection of Glucose Absorption, Lactic Acid Secretion, and ATP Level

When T24 and UMUC-3 cells were grown to the logarithmic growth phase, they were plated on six-well plates with 5 × 10^5^ cells per well. After 48 h of culture, the culture solution was collected, and the concentration of glucose and lactic acid as well as the level of ATP were determined by the biochemical automatic analyzer, respectively. Then, the cells were digested and counted after centrifugation. By subtracting the base values of glucose, lactic acid, and ATP in the culture fluid, the values of glucose production and lactic acid consumption as well as ATP level were calculated.

### Mice Model

First, T24 cells were infected with the indicated lentiviral vector and CNN1 (5 × 10^6^ cells per mouse in 200-μl volume). Then, T24 vector and T24-CNN1 were subcutaneously into the left armpit of 6-week-old BALB/c nude mice. After 21 days, the mice were anaesthetized with 2%–3% isoflurane. Then, the mice were sacrificed. Then, the tumor was removed and weighed. All the animal assays were approved by the Animal Ethical and Welfare Committee of Tianjin Medical University.

### Immunohistochemistry

Paraffin-embedded sections were firstly deparaffinized and then incubated with rabbit polyclonal anti-Ki-67 (#12075, Cell Signaling Technology) or rabbit polyclonal anti-CNN1 (#17819, Cell Signaling Technology) primary antibody at 4°C overnight. After being washed by TBST three times, the sections were then incubated with horseradish peroxidase–conjugated goat anti-mouse antibody (#4414, Cell Signaling Technology). The sections were washed by TBST three times and the signal was tested utilizing DAB Substrate Kit. Images were gained utilizing a microscopy.

### Data Statistics

The experimental data were analyzed by utilizing SPSS 22.0 statistical and GraphPad Prism 8.0 analysis software. The comparison of the two groups was carried out by Student’s t-test. One-way analysis of variance (ANOVA) and a *post-hoc* Dunnett’s test were utilized to analyze the mean comparison among more than two samples. *P* < 0.05 was regarded as statistically significant.

## Results

### Low Expression of CNN1 Is Predicted as Poor Prognosis in BC

**Figure 1:**
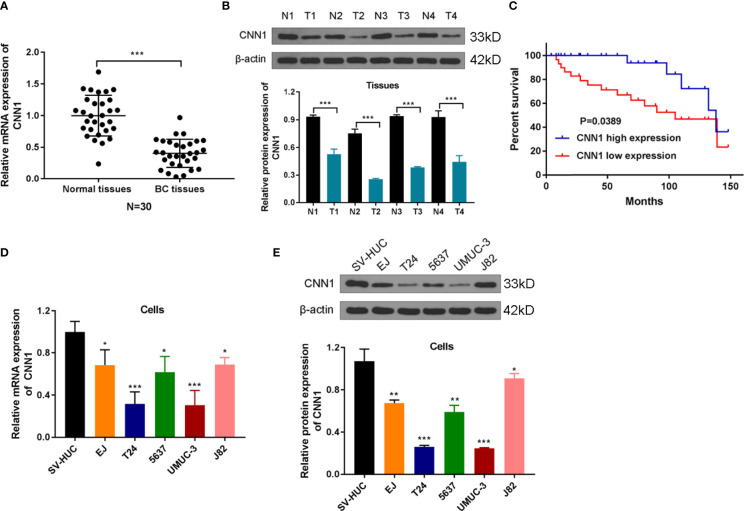
CNN1 is lowly expressed in BC tissues and cells. **(A)** The qRT-PCR experiment was utilized to test the mRNA expression level of CNN1 in the cancerous tissues and adjacent normal tissues of BC patients. ***p < 0.001 vs. normal tissues. **(B)** Western blotting was utilized to test the protein level of CNN1 in the cancerous tissues and adjacent normal tissues of BC patients. ***p < 0.001 vs. normal tissues. **(C)** Kaplan–Meier analysis was utilized to inquiry the OS of CNN1 in BC patients, which were determined by Log-rank test. **(D)** CNN1 mRNA expression level in SV-HUC, EJ, T24, 5637, UMUC-3, and J82 cell lines. *p < 0.001, ***p < 0.001 vs. SV-HUC. **(E)** CNN1 protein expression level in SV-HUC, EJ, T24, 5637, UMUC-3, and J82 cell lines. *p < 0.05, **p < 0.01, ***p < 0.001 vs. SV-HUC.

In order to probe the influences of CNN1 on BC, bioinformatics was used. First, the expression differences of CNN1 between BC and normal tissues were analyzed. The outcomes revealed that CNN1 was downregulated in BC tissues (n = 30) relative to normal tissues (n = 30; **Figure 1A**, *P* < 0.001). Then, Western blotting was utilized to test the relationship between CNN1 protein expression in BC patients and adjacent tissues. The outcomes indicated that the protein level of CNN1 was obviously lowly expressed in BC tissues (**Figure 1B**, *P* < 0.001). Then, we investigated the correlation of CNN1 expression and prognostic power in patients with BC. The consequences indicated that patients with low expression of CNN1 had a worse overall survival relative to those with high expression of CNN1 (**Figure 1C**, *P* = 0.0389). Then, the mRNA and protein expression of CNN1 in BC cell lines EJ, T24, 5637, UMUC-3, and J82 was detected. The human normal bladder epithelial cell line SV-HUC was used as a control. The results exhibited that the mRNA (**Figure 1D**) and protein (**Figure 1E**) levels of CNN1 were lowly expressed in both T24 and UMUC-3 cells. Thus, T24 and UMUC-3 cells were used in the subsequent analysis to overexpression CNN1. Altogether, these findings suggested that CNN1 was lowly expressed in BC tissues and cells; also, low expression of CNN1 could be considered as a prognostic factor in BC patients.

### Overexpression of CNN1 Suppresses the Property of BC Cells

**Figure 2:**
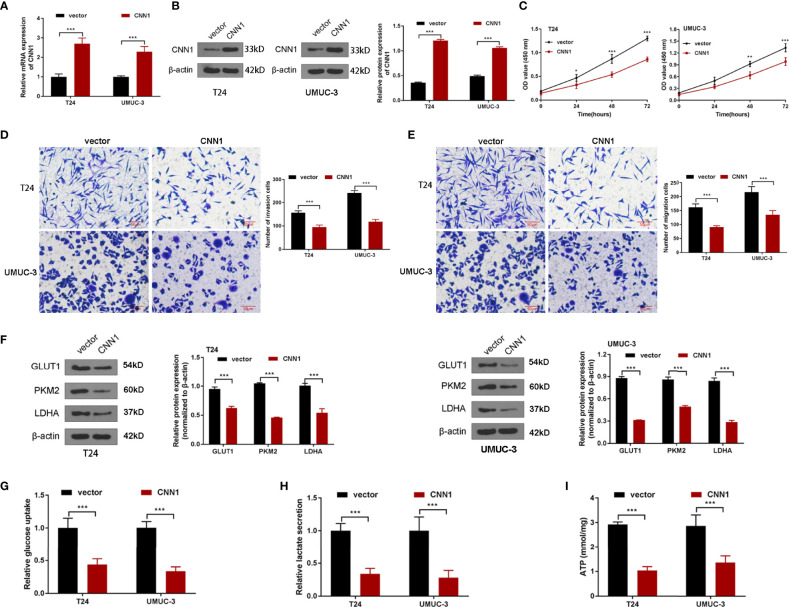
The influences of CNN1 on BC cell properties and glycolysis. **(A)** qRT-PCR was utilized to determine the mRNA expression of CNN1 in T24 and UMUC-3 cells. **(B)** Western blotting was utilized to test the protein level of CNN1 in T24 and UMUC-3 cells. **(C)** The influence of CNN1 overexpression on T24 and UMUC-3 cell proliferation was tested by CCK8 assay. **(D)** The influences of CNN1 overexpression on T24 and UMUC-3 cell invasion were determined by transwell assay (bar = 50 µm). **(E)** The impacts of CNN1 overexpression on T24 and UMUC-3 cell migration were determined by transwell assay (bar = 50 µm). **(F)** The protein levels of GLUT1, PKM2, and LDHA in T24 and UMUC-3 cells were tested by Western blotting. **(G)** The glucose consumption, **(H)** lactate production, and **(I)** ATP levels of T24 and UMUC-3 cells were tested by the corresponding kit.*p < 0.05, **p < 0.01, ***p < 0.001 vs. vector.

To probe the influences of CNN1 on BC cells, CNN1 expression was overexpressed in T24 and UMUC-3 cells by pcDNA3.1. The results of qRT-PCR and Western blotting demonstrated that pcDNA3.1 sequences visibly facilitated CNN1 expression in T24 and UMUC-3 cells, both in mRNA (**Figure 2A**) and protein levels (**Figure 2B**). Then, CCK8 and transwell were used to test the properties of BC cells. As illustrated in **Figures 2C-E**, overexpression of CNN1 repressed T24 and UMUC-3 cell proliferation, invasion and migration. Then, Western blotting was applied to further detect the impacts of CNN1 overexpression on the expression levels of glycolysis-related proteins GLUT1, PKM2, and LDHA. **Figure 2F** displayed that the protein levels of GLUT1, PKM2, and LDHA were all eased by CNN1 overexpression. Then, the kits were used to detect glucose absorption, lactate secretion, and ATP levels. (**Figures 2 G-I**) illustrated that, after overexpression of CNN1, the glucose consumption, lactate production, and ATP levels of T24 and UMUC-3 cells decreased (**Figures 2G-I**). Above all, overexpression of CNN1 eased the property of T24 and UMUC-3 cells.

### Overexpression of CNN1 Suppresses HIF-1α Signaling Pathway

**Figure 3:**
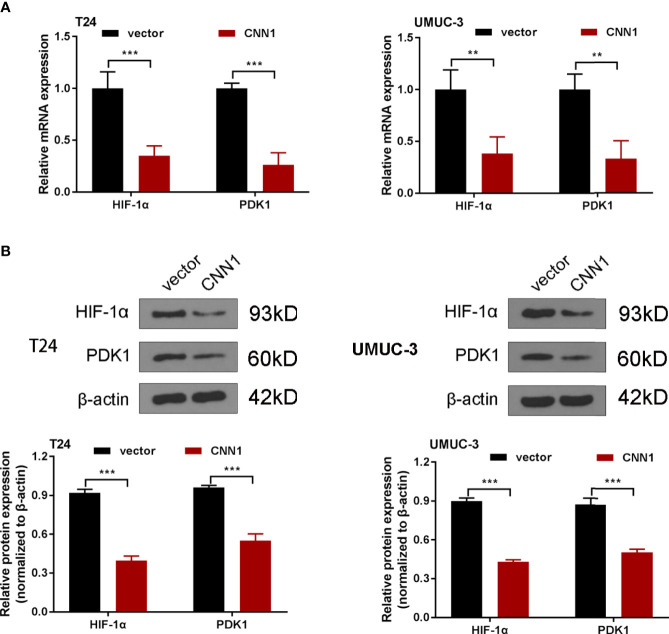
Overexpression of CNN1 suppresses HIF-1α signaling pathway. **(A)** qRT-PCR was utilized to test the mRNA expression of HIF-1α and PDK1 in T24 and UMUC-3 cells. **(B)** Western blotting was utilized to test the protein level of HIF-1α and PDK1 in T24 and UMUC-3 cells. **p < 0.01, ***p < 0.001 vs. vector.

It is believed that HIF-1α plays a significant role in the development of the BC (22). Besides, previous pieces of evidences have pointed out the association of CNN1 with HIF-1α in smooth muscle cell (21). For the purpose of exploring the mechanisms of CNN1 in BC, the impacts of CNN1 silence on HIF-1α pathway were determined. Western blotting was utilized to test the protein levels of HIF-1α and PDK1. As displayed in **Figures 3A, B**, overexpression of CNN1 suppressed the mRNA and protein levels of HIF-1α and PDK1 in T24 and UMUC-3 cells (**Figure 3**). All the above findings suggested that overexpression of CNN1 suppressed HIF-1α signaling pathway in BC.

### HIF-1α Activation Strengthened the Depressed Effect of CNN1 in BC Cells

**Figure 4:**
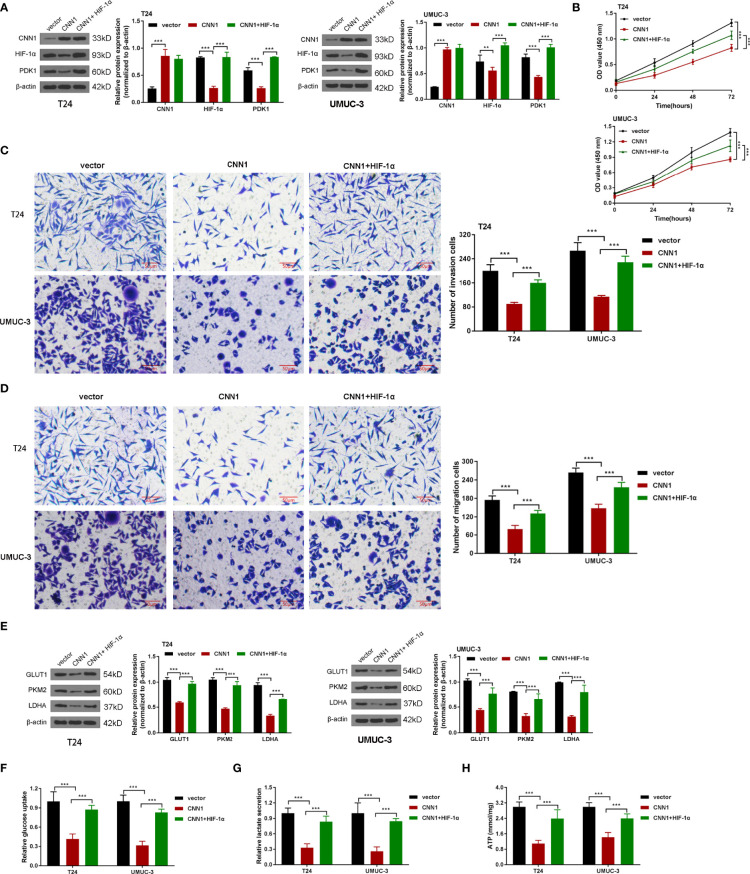
HIF-1α activation strengthened the depressed effect of CNN1 in BC cells. **(A)** The protein levels of CNN1, HIF-1α, and PDK1 were tested by Western blotting in T24 and UMUC-3 cells. **(B)** T24 and UMUC-3 cell proliferation was detected by CCK8 assay. **(C)** T24 and UMUC-3 cell invasion was tested by transwell assay (bar = 50 µm). **(D)** T24 and UMUC-3 cell migration was detected by transwell assay (bar = 50 µm). **(E)** The protein levels of GLUT1, PKM2, and LDHA in T24 and UMUC-3 cells were tested by Western blotting. **(F)** The glucose consumption, **(G)** lactate production, and **(H)** ATP levels of T24 and UMUC-3 cells were tested by the corresponding kit. **p < 0.01, ***p < 0.001 vs. vector.

To further manifest the mechanism of CNN1 function on BC cells, CNN1 overexpression combined with HIF-1α was cotransfected into T24 and UMUC-3 cells. Western blotting outcomes showed that HIF-1α activation accelerated the suppressed role of CNN1 overexpression on the protein levels of HIF-1α and PDK1 in T24 and UMUC-3 cells (**Figure 4A**). CCK8 results indicated that overexpression of CNN1 suppressed the T24 and UMUC-3 cell proliferation, and HIF-1α activation receded the repressed role of CNN1 on the proliferation of T24 and UMUC-3 cells (**Figure 4B**). Transwell assay results revealed that HIF-1α activation weakened the inhibitory impact of CNN1 on the invasion and migration of BC cells (**Figures 4C, D** **)**. Moreover, CNN1 overexpression suppressed the protein levels of GLUT1, PKM2, and LDHA, which were rescued by HIF-1α activation (**Figure 4E**). Furthermore, the decreased glucose consumption, lactate production, and ATP levels of T24 and UMUC-3 cells were promoted by HIF-1α activation (**Figures 4F-H**). Together, these results provide important insights into that HIF-1α activation enhanced the suppressive influence of CNN1 overexpression on cell proliferation, invasion, migration, and glycolysis in BC cells.

### Overexpression of CNN1 Represses the Growth of Xenograft Tumor in Nude Mice

**Figure 5:**
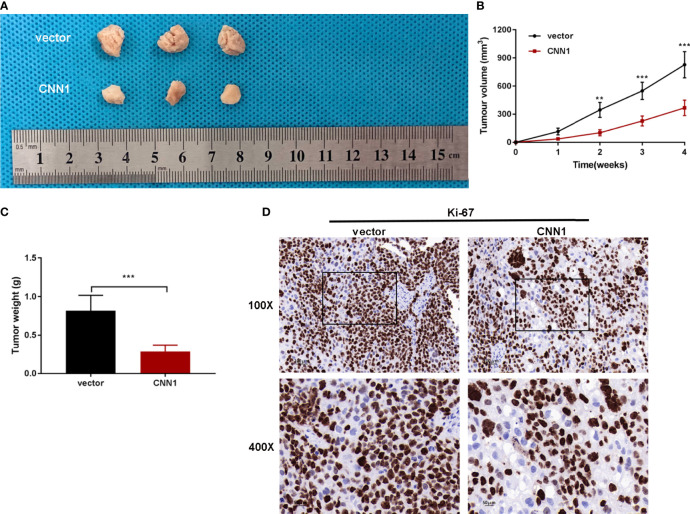
CNN1 overexpression suppresses BC cell growth *in vivo*. **(A)** Tumors were gathered from mice. **(B)** Tumor volume curve was analyzed. **(C)** Tumor weight was monitored. **(D)** Nude mouse tumor tissues were used for immunohistochemical experiments to detect the expression levels of Ki-67 and CNN1. **p < 0.01, ***p < 0.001 vs. vector.

In order to research the relationship between CNN1 and BC *in vivo*, subcutaneous xenograft tumor model in nude mice was built. T24-pcDNA3.1-CNN1 (CNN1) or T24-pcDNA3.1 (vector) cells were injected into mice. Xenograft tumor model with CNN1 revealed overtly lowered in tumor volume and weight relative to mice vector xenograft tumor model (**Figures 5A-C**). Nude mouse tumor tissues were used for immunohistochemical experiments to detect the expression levels of Ki-67 and CNN1. **Figure 5D** illustrated that CNN1 decreased Ki-67 in BC nude mice. Collectively, the above findings indicated that CNN1 overexpression could impede BC cell growth in nude mice.

## Discussion

In this article, it was found that CNN1 was lowly expressed in BC tissues and cell lines. Besides, a low expression of CNN1 might be associated with poor prognosis of BC patients. Moreover, overexpression of CNN1 alleviated the property and reduced the protein levels of glycolysis-related proteins GLUT1, PKM2, and LDHA, which might through HIF-1α pathway in BC cells.

Through bioinformatics analysis of the differentially expressed genes in BC, it was found that CNN1 was lowly expressed in BC tissue. In a reported BC bio-analysis article, it was also found that CNN1 was low in BC, and CNN1 is related to patient survival (11). However, there are no relevant reports on the effect of CNN1 on BC progression and metabolic reprogramming. In line with the above study, CNN1 was discovered to lowly express in BC tissue and lowly CNN1 was related to worse BC patients, so as to further verify the results of previous studies.

Moreover, this article discovered that overexpression of CNN1 alleviated the proliferation, invasion, and migration of BC cells. In hepatocellular carcinoma cells, CNN1 was a cancer inhibitor gene that turned into a marker of cell migration (12). It was demonstrated that CNN1 suppressed lung squamous cell carcinoma cell invasion and migration (23). Consistent with the above studies, we discovered that CNN1 overexpression lightened BC cell proliferation, invasion, and migration.

In this paper, it was discovered that overexpression of CNN1 reduced the protein expression levels of glycolysis-related proteins GLUT1, PKM2, and LDHA in BC cells. As one member of the glucose transporter family, GLUT1 modulates glucose transport across the cell membrane (24). Glucose transmembrane transport is not only the first step of glucose metabolism but also the rate-limiting step of glycolysis (24). There is one study indicated that SIRT1/GLUT1 axis accelerated BC progression through modulation of glucose uptake (25). The glycolytic phenotypes do not show homogeneously in cancers due to the diverse expression of PKM2 (26). In BC, the m5C amended in PKM2 mRNA in the HIF-1α/ALYREF/PKM2 axis might facilitate the glucose metabolism (27). LDH catalyzes the reversible conversion of pyruvate to lactic acid, which is the last step of glycolysis. The different combinations of the two subunits of LDHA and LDHB produce five LDH isoenzymes (28). Through STAT3/LDHA pathway–mediated glycolysis, PLCϵ facilitated urinary BC cell proliferation (29). In line with the above studies, in our paper, we discovered that CNN1 overexpression decreased the protein levels of GLUT1, PKM2, and LDHA in BC cells. Moreover, this paper discovered that overexpression of CNN1 alleviated the properties of BC cells, which were might through glycolysis-related HIF-1α pathway. However, the increased expression of HIF-1α and PDK1 may be only due to the cotransfection of HIF-1α, which was not sufficient to demonstrate that HIF-1α activation accelerated the suppressed role of CNN1. Further studies might be executed to prove this conclusion, which might be reported in future articles. Nevertheless, we discovered that CNN1 regulated the properties of BC cells partly through HIF-1α pathway.

In conclusion, the present paper discovered that CNN1 functions as a cancer repressor gene that inhibits BC cell proliferation and migration and represses glycolysis by regulating HIF-1α pathway. Meanwhile, CNN1-OE BC cells formed tumors in mice model, manifesting that CNN1 could serve as a potential marker for BC therapy.

## Data Availability Statement

The original contributions presented in the study are included in the article/supplementary materials. Further inquiries can be directed to the corresponding author.

## Ethics Statement

The studies involving human participants were reviewed and approved by the Medical ethics committee of The Second Hospital of Tianjin Medical University. The patients/participants provided their written informed consent to participate in this study. The animal study was reviewed and approved by the Animal Ethical and Welfare Committee of Tianjin Medical University. Written informed consent was obtained from the individual(s) for the publication of any potentially identifiable images or data included in this article.

## Author Contributions

ZZ and XL carried out the experiment and drafted the manuscript. SR and ZZ participated in the design of the study and performed the statistical analysis. WZ participated in its design and coordination and helped to draft the manuscript. All authors have read and approved the final manuscript.

## Funding

This work was supported by the Second Hospital of Tianjin Medical University Central Laboratory Research Fund Project (NO:2019ydey04).

## Conflict of Interest

The authors declare that the research was conducted in the absence of any commercial or financial relationships that could be construed as a potential conflict of interest.

## Publisher’s Note

All claims expressed in this article are solely those of the authors and do not necessarily represent those of their affiliated organizations, or those of the publisher, the editors and the reviewers. Any product that may be evaluated in this article, or claim that may be made by its manufacturer, is not guaranteed or endorsed by the publisher.
